# Outdoor open pond batch production of green microalga *Botryococcus braunii* for high hydrocarbon production: enhanced production with salinity

**DOI:** 10.1038/s41598-020-59645-5

**Published:** 2020-02-17

**Authors:** Suneerat Ruangsomboon, Jantra Dimak, Buppha Jongput, Itsanun Wiwatanaratanabutr, Pornthiwa Kanyawongha

**Affiliations:** 1 Program in Fisheries Science, Faculty of Agricultural Technology, King Mongkut’s Institute of Technology Ladkrabang, Bangkok, 10520 Thailand; 2 Center of Excellence in Applied Biosciences, King Mongkut’s Institute of Technology Ladkrabang, Bangkok, 10520 Thailand; 3 Industrial/University Collaborative Research Center (IUCRC), Faculty of Agricultural Technology, King Mongkut’s Institute of Technology Ladkrabang, Bangkok, 10520 Thailand; 4 Department of Plant Production Technology, Faculty of Agricultural Technology, King Mongkut’s Institute of Technology Ladkrabang, Bangkok, 10520 Thailand

**Keywords:** Field trials, Applied microbiology

## Abstract

The aim of this work was to enhance the biodiesel quality and hydrocarbon content of green microalga *B*. *braunii* strain KMITL 2 cultivated outdoor under several salinity conditions in a batch production. The enhancement would be such that the microalgal biodiesel qualities met or exceeded the current standard so that it would be a good raw material for biodiesel production. The microalga production was in 300 L open oval ponds, among various salinity levels tested (0–20 ppt), 5 ppt was the best for hydrocarbon production, yielding 54.2 ± 0.9% hydrocarbon content and 5.1 ± 0.4 g L^−1^ biomass. As the microalga production was scaled up by cultivation in 3,675 L open raceway pond under the 5 ppt condition, the microalga yielded a bit higher hydrocarbon content (58.8 ± 2.9%) but much lower biomass (2.5 ± 0.5 g L^−1^). The production in both oval and raceway ponds yielded a nearly identical biodiesel property (61.06–67.42 cetane number) which exceeded the value specified in international standards. Therefore, it was concluded that *B*. *braunii* strain KMITL 2 can be batch cultivated in an open pond at optimum salinity to yield sufficient hydrocarbon and biomass for biodiesel production.

## Introduction

Currently, the use of microalgae as raw material for biodiesel production, replacing dwindling fossil fuel, is attracting a lot of interest^1,2^ because of the following reasons: microalgae are not a human food, so using them for biodiesel production will not affect human food supply; microalgae production does not need fertile soil, so they will not cause a forest encroachment issue; and algal biodiesel has been tested on real diesel engines and found to be as good a fuel as fossil diesel^3^.

Among various algal species, *Botryococcus braunii* has been widely accepted as an alga that contains the highest hydrocarbon content, most suitable for biodiesel production. Many research studies have been conducted to find environmental factors that affect its distribution, to find a cultivation method that would yield high biomass at low cost, and to investigate various extraction methods and kinetics that would be most suitable for biodiesel production^1,4–6^. All of these attempts were towards making use of this alga for commercial biodiesel production. Besides the findings from those studies, it has also been found that variations of some laboratory factors such as light intensity, nutrient concentration, type of medium, cultivation time, and salinity increased the algal biomass and its hydrocarbon content^7–11^.

For commercial biodiesel production, an alga must be cultivated to yield high biomass, high hydrocarbon or lipid content, and good biodiesel qualities. *B*. *braunii* fits this prescription nicely; not only does it have high hydrocarbon content, its cetane (CN) value also exceeds the value specified in two international standards, a minimum of 51 and 47 specified by the European EN 14214 and American ASTM D6751, respectively^12,13^. From a previous study, it was found that *B*. *braunii* strain KMITL 2 had a high lipid and hydrocarbon content, as high as 54.70% and 36.82%, respectively^8,14,15^; its CN value was in the range of 44.42–65.39^13,15,16^, hence, this strain can be a good feedstock for biodiesel production.

Cultivation of alga in outdoor open pond can produce a large amount of biomass because an outdoor pond can be much larger than a laboratory pond and holding a larger volume of water. This kind of cultivation system is low cost and easy to maintain^16,17^. Nevertheless, many outdoor cultivation factors are not as controllable as laboratory cultivation factors. In addition, whether growing *B*. *braunii* in an outdoor open pond would be suitable for biodiesel production had to be determined; by measured the hydrocarbon content, biomass production, and its basic biodiesel qualities, including cetane number, oxidative stability, iodine number, and cold-flow properties^18^, to see whether these properties met the international standards.

Other components of the biochemical composition were also needed to be determined, such as pigments, proteins, and carbohydrates, in order to plan out how to use the algal residue after hydrocarbon extracted, so that an investment in an outdoor cultivation would yield the highest return. For instance, the residue can be further used for bioethanol or biomethane production or used as an animal feed.

Although cultivation of *B*. *braunii* in an outdoor open pond was often contaminated by other algal species, some previous studies have shown a successful outdoor cultivation of this alga. For instance, Ranga Rao *et al*.^19^ showed a successful outdoor cultivation of this alga in a 40 L circular and raceway pond; Wan *et al*.^20^ showed the same in a 120 L open circular ponds; and Ashokkumar^21^ also showed the same in a 5000 L open raceway pond. Even though one of our previous studies showed that an appropriate level of salinity could increase the biomass and hydrocarbon content of *B*. *braunii* cultivated in laboratory^8^, there has been no report to date on the effect of salinity on the cultivation of this alga in a raceway pond.

Our preliminary study had shown that a cultivation of *B*. *braunii* in an open raceway pond supplied with flue gas from an electric power plant (Bang Pakong, Thailand) increased its biomass and reduced the cost of algal production. This power plant was located near Bang-Pakong River which was the source of freshwater for the algal cultivation. During the summer season, the salinity of water in this river varied from 0–18 ppt, which surely must have affected the algal cultivation, but its effect on *B*. *braunii* cultivation in open raceway pond was not investigated thoroughly.

From a previous report, only 5 ppt salinity level was investigated in *B*. *braunii* KMITL 2 cultivation in a small open pond, but no other salinity levels were tested^16^. The contributions from the previous study to this study are the following: (1) the range of tested salinity levels was expanded 4 times higher; and (2) the capacity of the pond was extended to over 10 times. In this study, various salinity levels (0–20 ppt) were tested under the same other conditions to find the optimum salinity for outdoor cultivation. After the optimum salinity has been determined, the production of this algal strain in a large open raceway pond under this optimum salinity would be investigated. The findings would be used to properly manage the algal production at Bang Pakong power plant.

The aims of this research were to determine the biomass, hydrocarbon content, and biodiesel qualities of *B*. *braunii* KMITL 2 cultivated in outdoor open ponds (a small oval pond and a raceway pond) under various levels of salinity. The optimum salinity level was expected to stimulate a higher algal production, and the production cost could be lowered by using natural sea water or salty water waste from a marine animal farm as a salinity source.

## Results and Discussion

### Effects of salinity on growth and hydrocarbon content of *B*. *braunii* KMITL 2 cultivated outdoor in a small, open oval pond

**Figure 1:**
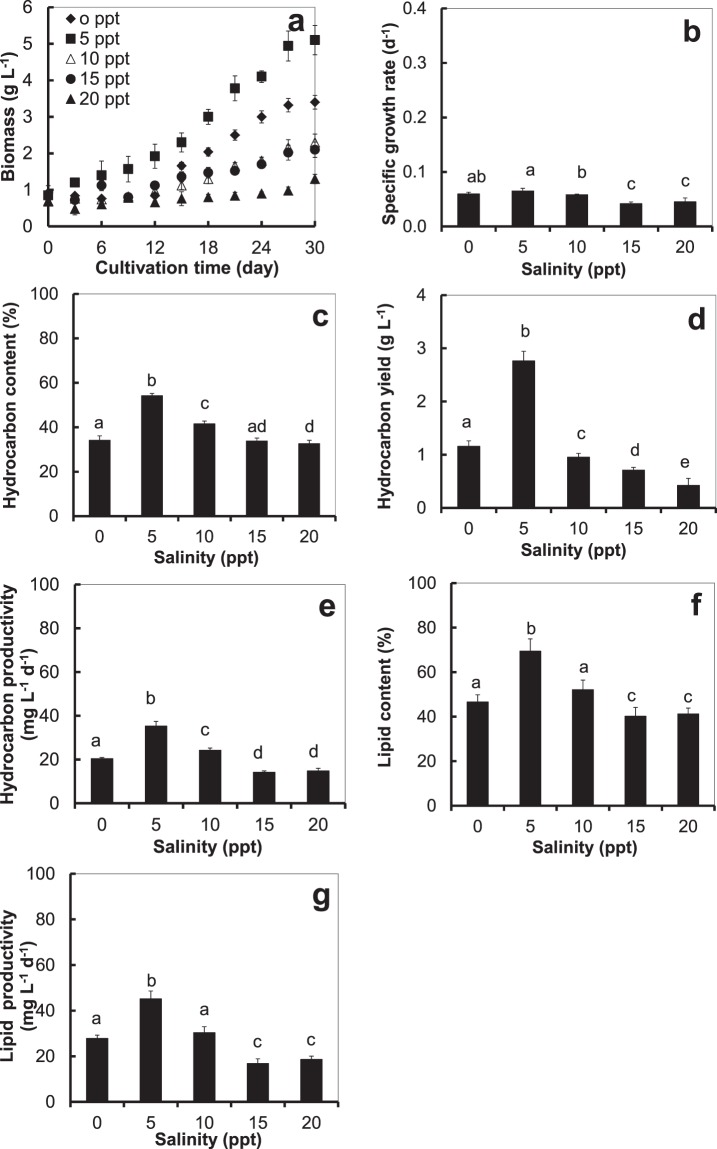
*B*. *braunii* cultivated outdoor in a small open pond at various levels of salinity, (**a**) biomass, (**b**) specific growth rate, (**c**) hydrocarbon content, (**d**) hydrocarbon yield, (**e**) hydrocarbon productivity, (**f**) lipid content, and (**g**) lipid productivity; different small letters (**a**–**c**, …) on the bars denote a significant difference (*p* < 0.05); Error bars are ± S.D. of four replicates.

At the end of the cultivation, the highest biomass yield and specific growth rate were found to be 5.1 ± 0.4 g L^−1^ and 0.065 ± 0.005 d^−1^ at 5 ppt salinity level, followed by 0, 10, 15, and 20 ppt in that order (Fig. 1a,b). Cultivation under this 5 ppt optimum salinity condition also gave the significantly highest (*p* < 0.05) hydrocarbon content, hydrocarbon yield, hydrocarbon productivity, lipid content, and lipid productivity at 54.2 ± 0.9%, 2.76 ± 0.18 g L^−1^, 35.29 ± 2.10 mg L^−1^ d^−1^, 69.43 ± 5.55% and 45.22 ± 3.45 mg L^−1^ d^−1^, respectively (Fig. 1c–g). These findings agree well with a finding by Hu^22^ that an increase in salinity resulted in a slight increase in total hydrocarbon production of algae.

Despite our investigated alga being a freshwater alga, it thrived in Chlorella medium at 5 ppt salinity level, probably due to the seawater containing more minerals and nutrients (e.g., Cl^−^, Na^+^, Mg^2+^, Ca^2+^, K^+^) that promoted rapid growth. However, instead of promoting further growth, too much salinity stressed the alga by disrupting the balance of its intracellular water and ions^23^ and forced it to expend additional energy to restore the balance rather than to sustain growth.

When cultivated in a laboratory, this algal strain was reported to yield the highest biomass at 5 ppt salinity level^8^, but the biomass yield was much higher (5 times) when cultivated outdoor. This might be due to the higher outdoor temperature at 26–35 °C than the fixed indoor temperature at 25 °C and the stronger outdoor natural light at 814–1478 μE m^−2^ s^−1^ than the indoor light which was kept fixed at 200 μE m^−2^ s^−1^. This *B*. *braunii* KMITL 2 provided more biomass under a high temperature and strong light of an outdoor environment, in agreement with a finding by Bazaes^24^, who cultivated *B*. *braunii* LB572 outdoor in a pilot-scale reactor, that solar radiation had a major impact on the alga’s growth and biomass yield.

### Biomass and biochemical composition of *B*. *braunii* KMITL 2 cultivated outdoor under an optimum salinity condition in an open raceway pond

**Figure 2:**
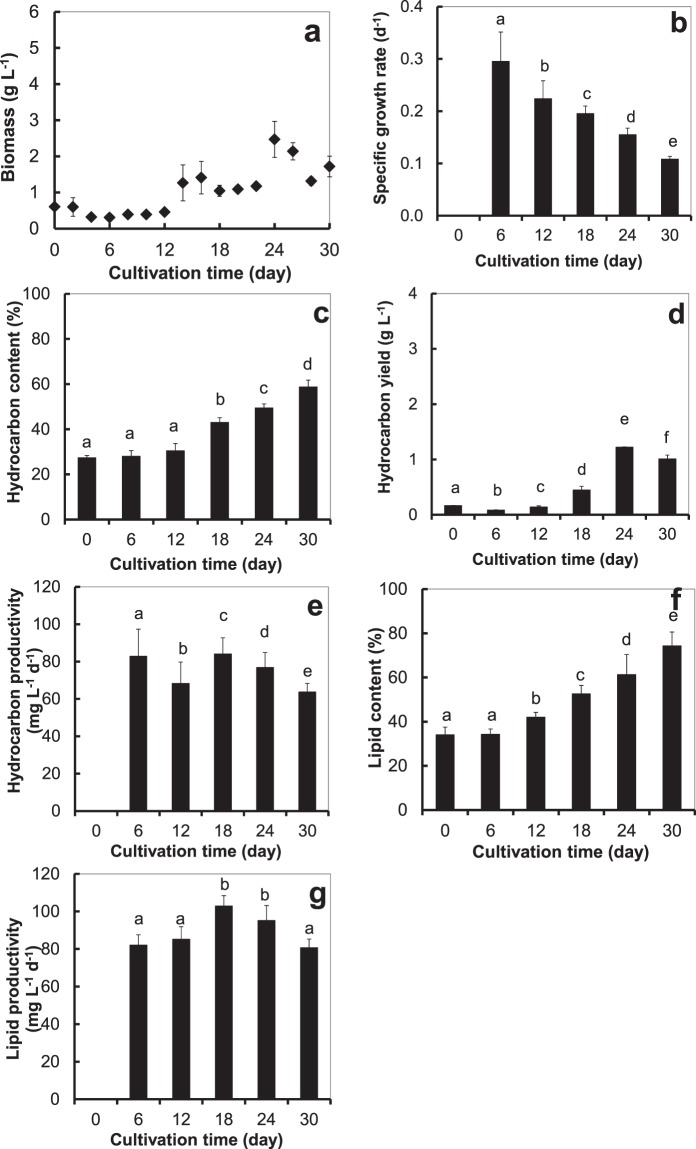
*B*. *braunii* cultivated outdoor in a large, open raceway pond at an optimum salinity level (5 ppt): (**a**) biomass, (**b**) specific growth rate, (**c**) hydrocarbon content, (**d**) hydrocarbon yield, (**e**) hydrocarbon productivity, (**f**) lipid content, and (**g**) lipid productivity; different small letters (**a**–**c**, …) on the bars denote a significant difference (*p* < 0.05); Error bars are ± S.D. of four replicates.

Besides cultivation in a small, 300 L open oval pond, the alga was also cultivated in a large 3,675 L open raceway pond under the same 5 ppt optimum salinity, and its growth and biochemical composition were monitored. The alga exhibited the highest biomass yield of 2.5 ± 0.5 g L^−1^ (Fig. 2a) on day 24^th^ of cultivation. There has been a report that a cultivation of *B*. *braunii* AP103 in an open raceway pond yielded 1.8 ± 0.1 g L^−1^ biomass^25^.

The alga exhibited the significantly highest (*p* < 0.05) specific growth rate of 0.30 ± 0.06 d^−1^ (Fig. 2b) after the 6^th^ day of cultivation. Beyond the 6^th^ day, the specific growth rate decreased, probably due to depletion of and competition for nutrients in the medium. In contrast, the hydrocarbon content, hydrocarbon yield and lipid content increased steadily throughout the experimental period (Fig. 2c,d,f), the highest being 58.8 ± 2.9%, 1.22 ± 0.01 g L^−1^, and 74.39 ± 6.20%, respectively. That steady increase might be due to nutrient depletion, forcing the alga to produce more hydrocarbon from photosynthesis^26^. The highest hydrocarbon and lipid productivity were 84.09 ± 8.59 and 102.86 ± 5.53 mg L^−1^ d^−1^ on the 18^th^ day of cultivation (Fig. 2e,g).

Outdoor cultivation in the small oval pond (Fig. 1a) gave 2.04 times higher biomass yield than cultivation in a large raceway pond (Fig. 2a), and even though the hydrocarbon content was 4.6% lower (Figs. 1c and 2c), the hydrocarbon yield was 2.73 times higher because of the much higher biomass yield (Figs. 1d and 2d). The water level in both the small open pond and in the large raceway pond was maintained at 35 cm, and the water circulation rate was the same at 0.17 m s^−1^. At the time of the experiment, the temperature and light intensity were slightly higher in the cultivation of the alga in the raceway pond. The reason that the alga yielded a higher biomass in the small open pond may be that the water that flowed away from the centre of the small oval pond got to the wall of the pond much quicker, since it was much smaller, and reflected back quicker, so the alga got circulated and exposed to light more thoroughly, hence it grew better. Thus, the higher biomass in the small oval pond cause from the algal cells got exposed to light for a longer time and more often, indicating that when cultivating this alga in a raceway pond, light is an important factor that must be considered.

Normally, *B*. *braunii* strain KMITL 2 is a colonial alga, however, when it was grown outdoor in the small oval pond and the large raceway pond for 14 days, single cells of this alga appeared on the pond surface and steadily increased, reaching 2 percent of total biomass at the end of the experiment (30 days). In addition, the longer the cultivation time, the more the single cells were produced, and they were difficult to harvest.

**Figure 3:**
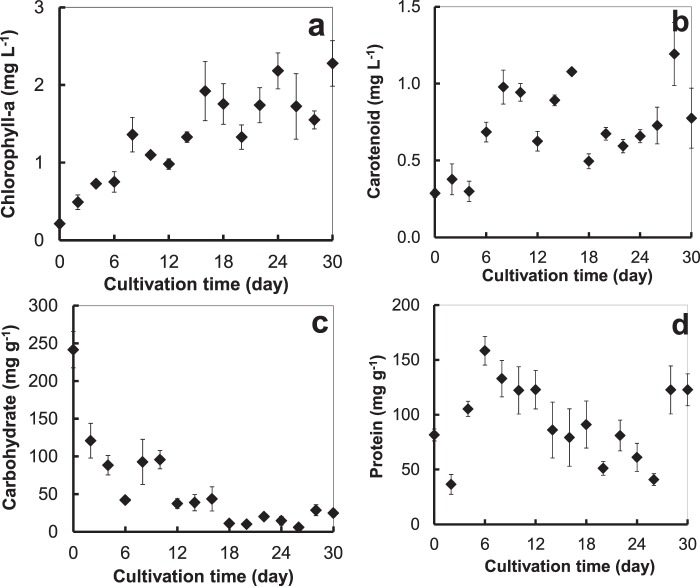
Chlorophyll-a (**a**), carotenoid (**b**), carbohydrate (**c**), and protein (**d**) contents of *B*. *braunii* cultivated in a large, open raceway pond at the optimum level of salinity.

The chlorophyll-a and carotenoid contents of the alga cultivated in the large, raceway pond increased with cultivation time. The highest levels were 2.27 ± 0.29 and 1.19 ± 0.20 mg L^−1^ found on the 30^th^ and 28^th^ day of cultivation, respectively (Fig. 3a,b). The highest carbohydrate content was 241.46 ± 24.00 mg g^−1^ (24.1%). This content decreased with cultivation time (Fig. 3c), as opposed to the steadily increased hydrocarbon content throughout the experiment (Fig. 2c), which might be because as the level of nutrients decreased, this alga converted carbohydrate to hydrocarbon^26^. The protein content varied unpredictably throughout the cultivation period, the highest being 158.35 ± 13.06 mg g^−1^ (15.8%) at 6 days after cultivation (Fig. 3d). A previous study reported that carbohydrate and protein contents of *B*. *braunii* AP103 cultivated in open raceway pond were 18 ± 0.92 and 17 ± 0.87%, respectively^25^.

The findings from this study indicate that *B*. *braunii* strain KMITL 2 can be cultivated outdoor, both in a small oval pond and a large raceway pond. During the 30 days of cultivation, no contamination from other microalgae was found. Some ciliate protozoa were found, but they did not consume much of the microalga, probably because the colonies of the microalga were too big as a food item for the protozoa.

Cultivation of this algal strain in a 5 ppt medium gave higher biomass (1.5 times) compared to the control. The cost of high-salinity seawater was 0.17 Baht per liter of medium (1 USD = 30.78 Baht). The average production cost per kilogram of biomass was 147 and 131 Baht when cultivated under 0 and 5 ppt, respectively. Overall, the production cost per kilogram of biomass was lower for 5 ppt medium.

### Fatty acid methyl esters (FAME) profile of *B*. *braunii* KMITL 2

The most frequently found fatty acid in outdoor cultivated *B*. *braunii* in a small, open oval pond was palmitic acid methyl ester (C16:0) (Table 1). It was found in every sample of *B*. *braunii* cultivated under 0–20 ppt salinity condition. The relative percentage of the palmitic acid methyl ester to the total FAME was around 23.14–49.02%, and the highest was found in the alga cultivated under 15-ppt salinity. In general, the alga cultivated under any salinity condition showed a higher percentage of C16:0 than the alga cultivated under no salinity condition. In other words, the alga cultivated under any salinity condition exhibited lower percentages of capric acid methyl ester (C10:0), undecanoic acid methyl ester (C11:0), lauric acid methyl ester (C12:0) and tridecanoic acid methyl ester (C13:0) than the alga cultivated under no salinity condition. The saturated and unsaturated fatty acid methyl esters from all treatments were 74.71–81.21% and 18.79–26.42%, respectively. The trend seems to be that cultivation of alga under a salinity condition yielded higher percentages of C16–18 (64.86–74.76%) than its cultivation under no salinity condition (44.72%). The percentage of FAME C10:0–C18:1 from all treatments were 86.07–95.16%.

**Table 1:** Fatty acid methyl ester (FAME) profiles of outdoor cultivated *B*. *braunii* in a small, open oval pond at various salinity levels for 30 days.

Fatty acid (%)	Salinity (ppt)				
	0	5	10	15	20
Butyric Acid (C4:0)	0	0.23	0.01	0	0
Caproic Acid (C6:0)	0.20	0.15	0.11	0.23	0.13
Caprylic Acid (C8:0)	0	0.27	0.13	0.23	0.29
Capric Acid (C10:0)	1.26	0.71	1.12	0.51	0.60
Undecanoic Acid (C11:0)	17.82	11.06	10.08	10.97	9.85
Lauric Acid (C12:0)	20.24	11.79	10.92	5.94	11.91
Tridecanoic Acid (C13:0)	9.01	5.04	5.92	2.25	4.82
Myristic Acid (C14:0)	1.80	1.60	1.42	1.78	1.66
Myristoleic Acid (C14:1)	1.91	1.35	1.47	1.69	1.24
Pentadecanoic Acid (C15:0)	0.74	0.60	0.64	0.46	0.74
cis-10-Pentadecenoic Acid (C15:1)	0.48	0.41	0.43	0.46	0.48
Palmitic Acid (C16:0)	23.14	31.79	34.59	49.02	34.45
Palmitoleic Acid (C16:1)	4.11	5.10	3.91	5.89	5.27
Heptadecanoic Acid (C17:0)	5.40	6.13	3.63	4.63	6.09
cis-10-Heptadecenoic Acid (C17:1)	2.90	0	0	0	2.97
Stearic Acid (C18:0)	1.12	6.67	4.52	3.78	3.96
Elaidic Acid (C18:1n9t)	1.12	2.10	1.77	1.88	1.85
Oleic Acid (C18:1n9c)	1.95	4.70	5.65	5.90	4.85
Linolelaidic Acid (C18:2n6t)	0.30	0	0.40	0.38	0.35
Linoleic Acid (C18:2n6c)	0.38	0.17	5.59	0.28	0.40
Linolenic Acid (C18:3n3)	3.63	6.60	3.64	2.15	6.19
Y-Linolenic Acid (C18:3n6)	0.67	1.60	1.45	0.85	1.27
cis-11-Eicosenoic Acid (C20:1)	0	0.34	0.26	0.26	0.23
cis-11,14-Eicosadienoic Acid (C20:2)	0	0.07	0.27	0	0
cis-11,14,17-Eicosatrienoic Acid (C20:3n3)	0	0.26	0.35	0.01	0.10
cis-8,11,14-Eicosatrienoic Acid (C20:3n6)	0	0.18	0.18	0.06	0
Arachidonic Acid (C20:4n6)	0.78	0.18	0.42	0.04	0.09
cis-5,8,11,14,17-Eicosapentaenoic Acid (C:20:5n3)	0.56	0.11	0.09	0.03	0
Heneicosanoic Acid (C21:0)	0.12	0.10	0.14	0	0.11
Behenic Acid (C22:0)	0.36	0.19	0.27	0.11	0.05
Erucic Acid (C22:1n9)	0	0.41	0.54	0.13	0
Lignoceric Acid (C24:0)	0	0.09	0.08	0.08	0.05
Saturated fatty acid	81.21	76.42	73.58	79.99	74.71
Unsaturated fatty acid	18.79	23.58	26.42	20.01	25.29
Monounsaturated fatty acid	12.47	14.41	14.03	16.21	16.89
Polyunsaturated fatty acid	6.32	9.17	12.39	3.80	8.40
C16–C18	44.72	64.86	65.15	74.76	67.65
C10:0–C18:1	93.00	89.05	86.07	95.16	90.74
Polyunsaturated fatty acid (≥4 double bond)	1.34	0.29	0.51	0.07	0.09

The FAME composition of the outdoor cultivated *B*. *braunii* at an optimum salinity level in a large, open raceway pond for a period of 30 days were as follows: the most frequently found FAME throughout the cultivation period was palmitic acid methyl ester (48.89–72.11%) (Table 2). The saturated and unsaturated fatty acid methyl ester were 74.57–83.00% and 17.00–25.43%. The percentages of FAME C16-18 and C10:0–C18:1 were 60.96–83.68% and 63.79–87.80%.

**Table 2:** Fatty acid methyl ester (FAME) profiles of outdoor cultivated *B*. *braunii* in a large, open raceway pond at an optimum salinity level 5 ppt for 30 days.

Fatty acid (%)	Cultivation time (day)					
	0	6	12	18	24	30
Butyric Acid (C4:0)	0	0	0	0.39	0	0
Caproic Acid (C6:0)	0	0	0	0.29	4.25	22.18
Caprylic Acid (C8:0)	0.50	0	0.03	0.50	0	0.22
Capric Acid (C10:0)	0	0.13	0.09	0.47	0.38	0.25
Undecanoic Acid (C11:0)	0.98	0.12	0.06	0.50	0.43	0.20
Lauric Acid (C12:0)	0	0.20	0.09	0.07	0.28	0.20
Tridecanoic Acid (C13:0)	1.30	0.70	0.05	0.08	0.35	0.31
Myristic Acid (C14:0)	4.81	2.84	1.99	2.22	2.54	0.82
Myristoleic Acid (C14:1)	1.76	0.43	0.24	0.95	0.43	0.47
Pentadecanoic Acid (C15:0)	2.47	1.24	0.71	0.64	0.90	0.46
cis-10-Pentadecenoic Acid (C15:1)	1.97	1.21	2.58	1.94	2.25	0.94
Palmitic Acid (C16:0)	52.35	68.05	72.11	65.63	61.55	48.89
Palmitoleic Acid (C16:1)	0.67	0.83	2.25	1.20	2.22	2.13
Heptadecanoic Acid (C17:0)	1.89	0.62	0.92	0.58	1.16	0.36
cis-10-Heptadecenoic Acid (C17:1)	0.50	0.23	0.33	2.04	1.38	0.11
Stearic Acid (C18:0)	8.91	3.77	3.64	2.38	2.73	2.07
Elaidic Acid (C18:1n9t)	0.83	1.90	1.59	0.63	1.10	3.94
Oleic Acid (C18:1n9c)	0.84	0.49	1.15	1.35	1.20	2.64
Linolelaidic Acid (C18:2n6t)	0.46	0.62	0.82	0.11	0.36	0.63
Linoleic Acid (C18:2n6c)	0.46	0.22	0.44	0.36	0	0.19
Linolenic Acid (C18:3n3)	0	0.20	0	0	0	0
Y-Linolenic Acid (C18:3n6)	0	0.20	0.43	0	0	0
Arachidic Acid (C20:0)	1.01	0.23	0.68	0.39	0	0
cis-11-Eicosenoic Acid (C20:1)	0.88	1.51	0	0.47	0	0
cis-11,14-Eicosadienoic Acid (C20:2)	3.52	1.15	1.45	0.81	0.15	0
cis-11,14,17-Eicosatrienoic Acid (C20:3n3)	0.60	0	0	0	0.20	0
cis-8,11,14-Eicosatrienoic Acid (C20:3n6)	0	1.03	0.59	0.50	0	0
Arachidonic Acid (C20:4n6)	1.03	0	0	0	0	0
Heneicosanoic Acid (C21:0)	4.84	0.35	0.21	0.20	0	0.64
Behenic Acid (C22:0)	1.01	1.98	1.68	0.83	0	0.37
Erucic Acid (C22:1n9)	0.50	0	0	0	0.40	0
cis-13,16-Docosadienoic Acid (C22:2)	2.62	5.75	3.38	7.50	12.18	9.56
cis-4,7,10,13,16,19-Docosahexaenoic Acid (C22:6n3)	1.43	1.86	0.68	0	0	0
Tricosanoice Acid (C23:0)	0	0	0	0	0	0.47
Lignoceric Acid (C24:0)	1.00	0	0.74	0.32	0	0
Nervonic Acid (C24:1)	0.86	2.14	1.07	6.65	3.56	1.95
Saturated fatty acid	81.07	80.23	83.00	75.49	74.57	77.44
Unsaturated fatty acid	18.93	19.77	17.00	24.51	25.43	22.56
Monounsaturated fatty acid	8.81	8.74	9.21	15.23	12.54	12.18
Polyunsaturated fatty acid	10.12	11.03	7.79	9.28	12.89	10.38
C16-C18	66.91	77.13	83.68	74.28	71.70	60.96
C10:0-C18:1	79.28	82.77	87.80	80.69	78.90	63.79
Polyunsaturated fatty acid (≥4 double bond)	2.46	1.86	0.68	0	0	0

Our findings of *B*. *braunii*’s FAME composition cultivated in both a small oval pond and a large raceway pond agree well with those reported in a previous work. Palmitic acid (C16:0) was the major fatty acid found in *B*. *braunii*, as reported by Ranga Rao *et al*.^7^ and Fang *et al*.^27^, and in green microalga *Chlorella vulgaris*^28^. However, Yoo *et al*.^29^ reported that C18:1 was the major component of *B*. *braunii* and C18:0 was the major fatty acid found in green microalga *S*. *dimorphus* KMITL^30^, while the major fatty acid of vegetable oil was C18^31^.

Fatty acid methyl esters (FAME) affect biodiesel qualities. The European standard (EN 14214) of FAME for diesel engines specifies that the linolenic acid methyl ester content must not exceed 12%^32^. In all treatments, the KMITL 2 strain passed this criterion. Another criterion was that the content of polyunsaturated, ≥4 double bonds, must not exceed 1%. The strain KMITL 2 cultivated in salinity 5–20 ppt also passed this criterion (Table 1). Cultivated in an open raceway pond under 5 ppt salinity, this alga passed the criterion after the 18^th^ day of cultivation (Table 2).

The strain KMITL 2 showed a higher percentage of saturated fatty acid than unsaturated fatty acid. A high percentage of saturated fatty acid can result in poor cold flow properties because the fatty acid tends to crystallize at high temperature, resulting in clogged fuel filters and pipes and inconsistent engine operation^33^. This study shows that the saturated fatty acid decreased when the salinity level increased (Table 1). Thus, cultivation of this algal strain under an optimum salinity level is a good way to reduce the saturated fatty acid content that causes poor cold flow properties.

The strain KMITL 2 cultivated under a high salinity level showed a higher percentage of C16–18 than the control (0 ppt salinity) (Table 1). Thus, it means that cultivating this strain in an optimum salinity medium produces a good biodiesel feed stock because the higher percentage of FAME C16–18 makes the hydrocarbon from the alga better for biodiesel production, as a higher proportion of FAME C16–18 provides a higher cetane number and combustion heat^34^.

FAME C10:0–C18:1 has a cetane number higher than 47^35^; alga cultivated in the raceway pond had high contents of C10:0–C18:1, 63.79–87.80% of total FAME. This FAME composition indicated that the strain KMITL 2 cultivated in an open raceway pond with 5 ppt salinity is suitable for use as biodiesel feedstock.

### Biodiesel qualities of outdoor cultivated *B*. *braunii* KMITL 2

Tables 3 and 4 show the biodiesel properties of *B*. *braunii* KMITL 2 cultivated in a small, open oval pond and a large, open raceway pond. The saponification value (SV) - a metric of the average molecular weight (or chain length) of all present fatty acids - of the alga cultivated in the small, oval pond was quite similar to the saponification value of the alga cultivated in the large, raceway pond, in the ranges of 217.96–234.13 and 200.03–249.81 mg KOH g^−1^, respectively. Normally, SV decreases when FAME with higher carbon length increases. The SV values of algal cultivated under 5–20 ppt salinity were lower than that of the alga cultivated in freshwater (0 ppt). The maximum limit for SV value in ASTM standard was 202 mg KOH g^−1^ ^36^. Only KMITL 2 cultivated in a raceway pond for 0–18 days passed this criterion. The alga in all treatments cultivated in the small oval pond showed a higher SV than the maximum limit, while the SV value of the alga cultivated in the large open raceway pond tended to increase with cultivation time.

**Table 3:** Biodiesel quality properties of outdoor cultivated *B*. *braunii* in a small, open oval pond under various levels of salinity.

Salinity (ppt)	SV (mg KOH g^−1^)	IV (g I_2_ 100 g^−1^)	CN	DU (wt.%)	LCSF (wt.%)	CFPP (°C)
0	234.13	28.73	62.29	25.11	3.42	−5.73
5	221.29	36.86	61.57	32.74	6.99	5.47
10	219.55	39.60	61.06	38.81	6.29	3.29
15	217.96	24.18	65.18	23.83	7.12	5.88
20	220.31	36.61	61.74	33.68	5.60	1.10

**Table 4:** Biodiesel quality properties of outdoor cultivated *B*. *braunii* in a large, open raceway pond throughout a 30-day period (under the optimum salinity level of 5 ppt).

Cultivation time (day)	SV (mg KOH g^−1^)	IV (g I_2_ 100 g^−1^)	CN	DU (wt.%)	LCSF (wt.%)	CFPP (°C)
0	200.69	30.76	65.65	29.54	14.20	28.13
6	200.03	30.65	65.77	30.78	11.89	20.87
12	201.87	23.20	67.42	24.77	13.71	26.58
18	201.61	26.55	66.60	33.79	10.03	15.03
24	209.26	30.30	64.65	38.70	7.52	7.15
30	249.81	25.74	61.58	32.93	6.49	3.90

The iodine values (IV) of the alga cultivated in the small oval pond and the raceway pond were nearly identical. IV is a metric of the total unsaturation of a biodiesel, which is connected to its oxidative stability^18^. Biodiesel with a low IV is more oxidatively stable than that of a higher one. The criterion of European standard for maximum IV is 120 g I_2_ 100 g^−1^. The IV values of outdoor cultivated *B*. *braunii* in a small oval pond under various salinity conditions were in the range of 24.18–39.60 g I_2_ 100 g^−1^, the lowest being of the alga cultivated under a 15 ppt salinity condition. There was no clear relationship between these IV values and the salinity levels. Similarly, the IV values of outdoor cultivated *B*. *braunii* in the large raceway pond were in the range of 23.20–30.76 g I_2_ 100 g^−1^ throughout the cultivation period, the lowest being of the alga cultivated for 12 days. These values varied unpredictably with cultivation time, but they were lower than those of other strains of *B*. *braunii* and several other strains^5,11,34,37^. The IV value of this algal strain is better than the IV value of *C*. *sosokiniana* and C. *vulgaris*, 133–160 g I_2_ 100 g^−1^ which did not pass the criterion^38^.

Outdoor cultivated *B*. *braunii* in a small oval pond under various salinity conditions exhibited high CN values. It exhibited the highest CN of 65.18 when cultivated under 15 ppt salinity level (Table 3), while the cultivated alga in the open raceway pond for a 30 day period exhibited the highest CN of 67.42 on the 12^th^ day (Table 4). It appears that the CNs had no clear correlation with the salinity level or cultivation time. CN is a metric of two biodiesel qualities: combustion quality and ignition delay time. A lower CN indicates poor ignition and engine performance. Based on two international standards^39,40^, the minimum CN should be at least 47 or 51. The CN values of *B*. *braunii* in all treatments were in the range of 61.06–67.42, far exceeding this threshold. The CN values of KMITL 2 strain were higher than those of other *B*. *braunii* strains (51.92–53.47^12^ and 55.4^5^). The CN values of green microalgae *C*. *sosokiniana* and *C*. *vulgaris* were 42.40 and 40.24, respectively^38^.

Degree of unsaturation (DU) is another indicator of oxidative and long-period storage stability—a diesel with a higher DU is less stable for long- period storage. DU has an important impact on biodiesel pour and cloud point - a higher DU resulting in better cold flow. The DU values of this outdoor cultivated algal strain cultivated for 30 days in a small oval pond under various salinity conditions and in a large raceway pond varied from 23.83–38.81 and 24.77–38.70%, respectively. These DU values were lower than those of green microalga *Chlorella vulgaris* at 65.49^28^ and at 69.03^41^ and lower than those of green microalgae *Scenedesmus obliquus* and *Chlorella pyrenoidosa* at 76.53–132.08%^37^. Cultivation of this algal strain in an outdoor open pond produced alga with similar CN values to those of alga cultivated in a laboratory (58.30–66.82), but its SV, IV and DU^14^ values were slightly lower.

Low long-chain saturated factor (LCSF) and cold-filter plugging point (CFPP) values indicate better low-temperature biodiesel properties^42^ - outdoor cultivation of *B*. *braunii* in both a small oval pond and a large raceway pond exhibited LCSF in the range of 3.42–7.12 and 6.49–14.20%, respectively and CFPP in the range of −5.73–5.88 and 3.90–28.13 °C, respectively. Unsaturated fatty acid composition exerts negligible effect on low-temperature properties that depend mostly on saturated fatty acid content^31^. Only the outdoor cultivated alga in the large raceway pond for less than 18 days exhibited a higher CPFF value than 20 °C, and the diesel produced by it may cause some clogging under low temperature. If this cultivation environment is chosen, further investigation and adjustment are needed. Various methods have been reported to improve CFPP value, such as enzymatic acidolysis, blending of biodiesel with conventional petrol diesel, use of chemical additives, and modification of fatty acid profile^43^.

Comparing the biodiesel properties of KMITL 2 strain with those of vegetable oils (palm, olive, peanut, rape, soybean, sunflower, grape, H.O. sunflower, almond, corn), the DU values of this algal strain were lower than those of vegetable oils (64.2–157.8), while the CFPP values were higher than vegetable oil (−12–10 °C). Most CN values of KMITL 2 strain were higher than those of vegetable oils (48–57), except palm oil (61)^31^.

It seems that, under the same optimum conditions, outdoor cultivation of the microalga in the small oval pond was better than in the large raceway pond (Fig. 2d). Given that their far-exceeding global standard biodiesel qualities were quite similar, outdoor cultivated *B*. *braunii* in the small oval pond provided 2.73 times higher hydrocarbon yield than that cultivated in the large raceway pond (Fig. 1d). The root of this discrepancy is likely to be the better water circulation offered by the small pond that resulted in less microalga settled down to the bottom of the pond and all of the microalga getting exposed to light more thoroughly and consequently, provided better growth and more biomass.

There was contamination from other green microalgae in the open pond after a long time of cultivation under 0–10 ppt salinity, but no contamination when the salinity was higher than 15 ppt. Thus, for the next study, long-term semi-continuous and continuous cultivation under 15 ppt salinity in a large open pond would be investigated to find out whether they would produce sufficient biomass for hydrocarbon production. In addition, the biodiesel properties of the hydrocarbons after a hydrocracking treatment should be determined.

## Conclusion

This study was investigate the cultivation of microalga *B*. *braunii* KMITL 2 under different levels of salinity, outdoor in small and large open ponds to enhance its biodiesel quality and biomass yield. Several factors affecting *B*. *braunii* production have been explored, but exploration of the salinity factor in productive cultivation of this microalga has not yet been done thoroughly. An exploration into this sensible factor is our contribution to the field.

The investigated alga, *B*. *braunii* KMITL 2, thrived in a low, 5 ppt salinity condition. For biodiesel production, the microalga cultivated in both a small open oval pond and a large raceway pond exhibited nearly identical biodiesel qualities, showing a cetane value of 61.06–67.42, far exceeding two international standards for biodiesel production. In addition, cultivation in a small, open oval pond gave a much higher hydrocarbon yield. Cultivated this algal strain in a salinity medium is a good method for reducing the saturated fatty acid content, that causes poor cold flow properties, and SV values. Cultivation of this alga in a high salinity (5–20 ppt) medium could reduce the percentage of polyunsaturated, ≥4 double bonds, to pass the criterion of European standard (EN 14214) of FAME for diesel engine. When cultivated in an open raceway pond under 5 ppt salinity, after 12 days of cultivation, the cultivated alga passed this criterion. All our findings indicate that *B*. *braunii* KMITL 2 can be cultivated outdoor in an open pond at optimum salinity for use as an alternative feedstock for biodiesel production.

## Materials and Methods

### Algal cultivation

*Botryococcus braunii* strain KMITL 2 was isolated from Klong Boat reservoir, Nakhon Nayok province, Thailand^8^. It was classified as *B*. *braunii* “A race” according to a hydrocarbon composition analysis^15^. The GenBank accession number was KX470608. The strain was grown in a Chlorella medium^44^, optimum for its hydrocarbon production and growth^14^, at 25 °C in a laboratory, i.e., in 1-L glass flasks under constant light from 200 μE m^−2^ s^−1^ fluorescent lamps and air bubbling. We used the culture as stock for all subsequent experiments.

### Effects of salinity on biomass, hydrocarbon production, fatty acid composition, and biodiesel quality of *B*. *braunii* KMITL 2 cultivated outdoor in a small open pond

In a preliminary test, some contamination from green microalgae (i.e. *Chlorella* sp. and *Chlamydomonas* sp.) were found after the 35^th^ day of cultivation and led to unsuccessful continuous and semi-continuous cultivation of *B*. *braunii* strain KMITL 2. Hence, in this study, experiments were conducted in a batch cultivation mode (for 30 days) of which cultivation time for each batch is much shorter, hence contamination from other microalgae could be controlled much better.

In preparing the cultivation medium, chemical nutrients of Chlorella medium were dissolved in water with a specified salinity level (0–20 ppt). The water was prepared by diluting high-salinity seawater (82 ppt) with dechlorinated tap water. The chloride, sodium, magnesium, calcium and potassium contents in the 82 ppt salinity seawater were 45.36, 25.34, 3.00, 1.02 and 0.98 g kg^−1^, respectively.

The microalga was cultivated in a Chlorella medium at 0, 5, 10, 15 and 20 ppt salinity levels in a 300-L oval fiberglass outdoor pond (0.90 × 1.19 × 0.35 m). The medium solution was continuously circulated at a rate of 0.17 m s^−1^, with air bubbling. The natural light intensity at the time of cultivation was 814–1478 μE m^−2^ s^−1^, and the temperature was 26–35 °C. The salinity level was monitored daily and dechlorinated tap water was added to maintain the specified salinity level. The effects of salinity levels on the biomass, hydrocarbon, fatty acid composition, and biodiesel quality of *B*. *braunii* were determined.

### Biomass and biochemical composition of *B*. *braunii* KMITL 2 cultivated outdoor under an optimum salinity condition in an open raceway pond

From a cultivation of *B*. *braunii* in a small 300 L open oval pond, it was found that 5 ppt salinity level resulted in the highest biomass and hydrocarbon yields of this algal strain. Hence, we attempted to cultivate it in a larger open raceway concrete pond, 1.5 × 7 m in width and length. The depth of the water in the pond was 0.35 m. The total volume of water was 3,675 L. A pump was installed horizontally on the bed of the pond. It circulated water continuously at a rate of 0.17 m s^−1^.

The alga was illuminated with an average of 839–1,513 μE m^−2^ s^−1^ natural light at a temperature between 26–36 °C. Samples of the alga were taken and analyzed for its biomass, hydrocarbon content, and fatty acid composition every 6 days until the stationary phase was reached (around 30 days). To find the relative contents of substances in its biochemical composition so that the leftover algal biomass after the hydrocarbon was extracted could be recycled, analyses of chlorophyll-a, carotenoid, carbohydrate, and protein were conducted every 2 days. The volume and salinity level of the water were strictly controlled. A proper amount of dechlorinated water was added daily to maintain the volume and the specified salinity level.

### Determination of algal biomass, pigment, carbohydrate, and protein contents

Ten milliliters of algal samples were filtered through a glass microfiber filter paper (GF/C, Whatmann) and washed with distilled water. Then, the collected algal cells were dried for 24 h at 105 °C and cooled down to room temperature in a desiccator. The dry biomass was weighed. A method described by Becker^45^ was used to determine its Chlorophyll-a and carotenoid contents. A phenol sulfuric acid method^46^ was used to analyze the carbohydrate content. The protein content was determined by a Lowry method^47^. The specific growth rate (μ) was calculated by an equation described by Ceron^48^.

### Extraction and analysis of total hydrocarbon, total lipid, and fatty acids

Algal samples were harvested with a nylon mesh filter (pore size 32 μm) and dried in a hot air oven at 40 °C then ground into powder with a mortar and pestle. Next, 500 mg of dried biomass was extracted with n-hexane, and the extracted mixture was sonicated with a Transonic model 460/H (Elma, Singen, Germany) at 70 Hz and at room temperature. The extraction procedure was repeated once, and the extracted mixture was subjected to silica gel (Silica gel 60, 230–400 mesh, Merck) column chromatography with n-hexane as the solvent for column preparation and the mobile phase. Clear elutes were collected, and the collection was stopped when a yellow elute of carotene reached the end of the column. The hydrocarbon extract was dried in a rotary evaporator and weighed. Then, samples were sent to a commercial laboratory, and their hydrocarbon composition was analyzed by GC-MS to confirm the race of this algal strain.

Lipids were extracted from 500 mg of dried biomass with a mixture of chloroform:methanol:water (1:2:0.8, v/v). Algal cells were sonicated. This extraction process was repeated twice. The extracted product was dried in a rotary evaporator and weighed. The fatty acids were converted to methyl esters by direct transmethylation, following the 965.49 AOAC official method (preparation of methyl esters of fatty acids)^49^. The esters were detected with an Agilent Technologies 6890 N Gas Chromatography system (USA) with a flame ionization detector. The details of all methods reported in this section have already been described in a previous work^30^.

### Determination of biodiesel qualities

Formulas reported by Ramos^31^, Francisco^34^, and Wu and Miao^37^ were used to determine various biodiesel qualities: saponification value (SV), iodine value (IV), cetane number (CN), degree of unsaturation (DU), long-chain saturated factor (LCSF), and cold filter plugging point (CFPP).

### Statistical analysis

Results are reported as mean and standard deviation of four replicates of each kind of readings. Significant differences at *p* < 0.05 were determined by Analysis of Variance (ANOVA).
